# Assessing cranial morphology for sex determination in juvenile alpine swifts (*Tachymarptis melba*)

**DOI:** 10.1038/s41598-026-41421-6

**Published:** 2026-02-24

**Authors:** Tomasz Szara, Ebuderda Günay, Buket Çakar, Nilay Tezsay, Kelvi Shehu, Berke Batmankaya, Ergün Bacak, Gökhan Gün, Ozan Gündemir

**Affiliations:** 1 Department of Morphological Sciences, Institute of Veterinary Medicine, Warsaw University of Life Sciences-SGGW, Warsaw, 02-776 Poland; 2 Department of Wild Animal Diseases and Ecology, Faculty of Veterinary Medicine, Istanbul University-Cerrahpasa, 34320 Istanbul, Turkey; 3 Institute of Graduate Studies, Istanbul University-Cerrahpasa, 34320 Istanbul, Turkey; 4 Forestry Vocational School, Istanbul University-Cerrahpasa, 34473 Istanbul, Turkey; 5 Department of Molecular Biology and Genetics, Bogazici University, 34342 Istanbul, Turkey; 6 Department of Anatomy, Faculty of Veterinary Medicine, Istanbul University- Cerrahpasa, 34320 Istanbul, Turkey

**Keywords:** Alpine swift, Cranial morphology, Sexual dimorphism, Geometric morphometrics, Anatomy, Ecology, Ecology, Evolution, Zoology

## Abstract

The cranial morphology of juvenile alpine swift (*Tachymarptis melba*), a species adapted for an aerial lifestyle, has received limited attention. This study examined skull shape and size variation in 100 juveniles (57 females, 43 males), collected from 2020 to 2024 at Istanbul University-Cerrahpaşa after natural death. Using 3D scanning and 18 landmarks, we applied Procrustes ANOVA and principal component analysis (PCA) to assess the effects of sex, centroid size, body weight, and cranial length. Sex significantly influenced skull shape but not size. Centroid size showed no significant effect on shape in females or males. Body weight significantly impacted shape, more so in males than in females, whereas cranial length had no effect. PCA revealed minor sex-based shape differences along PC1, though not statistically significant. These results indicate subtle sexual dimorphism in juvenile skull shape, driven partly by weight, with no variation in size. This work highlights the role of early developmental factors in rapid morphological development, setting the stage for further ontogenetic studies.

## Introduction

The alpine swift (*Tachymarptis melba*), a highly specialized aerial species, is renowned for its remarkable flight capabilities. It spends most of its life in flight while foraging, mating, and even sleeping^1^. These adaptations make swifts a compelling subject for studying morphological and physiological traits that support their unique lifestyle. Cranial morphology is crucial for feeding efficiency, sensory processing, and structural integrity during high-speed flight^2^. While adult swifts have been extensively studied for their ecological and behavioral adaptations^3–5^, juvenile swifts have received relatively little attention, particularly regarding cranial development and potential sexual dimorphism. Understanding these early morphological characteristics is essential, as they may underpin the specialized adaptations observed in adults and provide insights into developmental processes^6^. Age-related differences in biometrics and body condition, such as lower mass in second-year birds compared to adults, further highlight the significance of early developmental variation in this species^7^. Recent studies on cranial morphology in other animals, such as livestock guardian dogs and cats, highlight the utility of advanced morphometric techniques in detecting subtle variations, suggesting their applicability to avian species, like swifts^8,9^.

Sexual dimorphism, the phenotypic differentiation between males and females, is widespread in birds, manifesting in differences in size, shape, or plumage^10^. In swifts, where both sexes share a highly aerial lifestyle, dimorphism is expected to be subtle, especially in juveniles where growth and maturation are incomplete^6^. Skull morphology often reflects such differences, influenced by genetic, hormonal, or ecological factors^11^. Geometric morphometrics, a powerful tool for quantifying shape and size variation, has proven effective in detecting subtle morphological differences in birds^12^. For example, studies on quails have successfully employed geometric morphometrics to identify sex-based differences, providing a model for investigating swifts^13^. However, studies focusing on juvenile swifts remain limited, leaving gaps in our understanding of how cranial traits develop and diverge early in life.

Beyond sex, factors such as body weight and cranial length may influence skull morphology, particularly in a species where flight efficiency is paramount^14^. As an indicator of overall mass, body weight could affect cranial shape through biomechanical demands or growth-related changes. In contrast, cranial length may reflect adaptations in beak or braincase structure tied to feeding or sensory functions^15^. These factors are likely dynamic in juveniles, as rapid growth and environmental interactions shape their morphology before adulthood^16^. The interplay of these variables with cranial variation in swifts remains underexplored, yet it is critical for understanding how early development aligns with their aerial lifestyle. Morphometric analyses in mammals, such as sheep and foxes, demonstrate that body weight and cranial length can significantly influence skull shape, suggesting parallel investigations in swifts could yield insights^17,18^.

Recent advancements in 3D scanning technology and morphometric analysis offer precise tools to address these questions^19^. Techniques such as Procrustes analysis and principal component analysis (PCA) allow researchers to disentangle the contributions of size, shape, and external variables to morphological variation^20^. This study leverages these tools to examine cranial morphology in juvenile alpine swifts, focusing on 100 individuals collected between 2020 and 2024. The primary objectives are to assess whether skull shape and size differ between male and female juvenile swifts, evaluate the influence of centroid size on shape variation within each sex, and examine the effects of body weight and cranial length on cranial morphology. By focusing on juveniles, identified through distinct plumage characteristics and confirmed via molecular sexing^21^, this research aims to establish a baseline for understanding cranial development and early sexual dimorphism in swifts. The findings are expected to contribute to a broader understanding of avian developmental biology, highlight the adaptive strategies of this species, and inform future studies on the ontogenetic changes and ecological pressures that shape swift morphology.

## Materials and methods

### Animals

This study was conducted on 100 juvenile alpine swifts (*Tachymarptis melba*), consisting of 57 females and 43 males. The specimens were collected between 2020 and 2024 from individuals who were admitted to the Istanbul University-Cerrahpaşa and had died of natural causes. All birds were identified as juveniles based on distinct plumage characteristics. Juvenile swifts exhibit dark body feathers with white edges and thin white margins on median coverts (MC) and greater coverts (GC). The median and greater coverts are narrower and less rounded, and the fifth rectrix (R5) has a convex curve on both sides of the shaft. In contrast, adult swifts, which were excluded from the study, show worn body feathers with minimal light-colored areas at the feather tips during summer. Newly molted feathers have only a narrow pale band at the tip, while the median and greater coverts are broader and more rounded or nearly square-shaped at the tips. Additionally, the adults’ fifth rectrix (R5) is sharply pointed, with slightly concave irregularities on both sides of the shaft.

Body weight and cranial length measurements were recorded during the study. The body weight was measured using a digital balance (precision: 0.01 g), and the cranial length was determined from the beak tip to the posterior edge of the cranium using a digital caliper (precision: 0.01 mm).

### Sex determination

Genomic DNA was extracted from 1 to 2 mm segments of feather calamus from each individual using the Genomic DNA from Tissue Kit (Macherey-Nagel GmbH & Co. KG, Dueren, Germany), following the manufacturer’s instructions. The CHD-W and CHD-Z gene regions, commonly used for molecular sexing in birds, were amplified using the 2550 F and 2718R primers^21^. PCR reactions were performed in a 25 µL reaction mixture containing 1× Taq Buffer (Thermo Fisher Scientific, Waltham, MA, USA), 1 mM MgCl₂, 0.2 mM of each dNTP, 0.5 µM of each primer, 0.5 U Taq polymerase (Thermo Fisher Scientific), and 1–5 ng of genomic DNA. Amplifications were carried out in a t100 Biorad thermal cycler under the following conditions: an initial denaturation at 95 °C for 3 min, followed by 40 cycles of 95 °C for 30 s, 50 °C for 30 s, and 68 °C for 45 s, with a final extension step at 68 °C for 5 min. PCR products were separated on a 2% agarose gel and electrophoresed in TAE buffer at 100 V for 45 min to visualize the CHD-W and CHD-Z gene bands.

### Skull preparation, 3D scanning, and landmarks

The skulls were carefully boiled for approximately 20 min to soften the tissues and facilitate the removal of muscles and skin. Special care was taken due to the juvenile status of the specimens. To minimize potential shape deformation during boiling, we monitored the process closely and avoided excessive exposure. A pilot test was conducted on two additional juvenile swift skulls (not included in the main dataset). One skull was scanned after manual soft tissue removal (pre-boiling) and again after the 20-minute boiling procedure. Geometric morphometric comparison revealed no significant shape differences, indicating a minimal impact of boiling on cranial shape.

Following boiling, the skulls were soaked in a 25% hydrogen peroxide solution for 5 min to remove fatty tissues. Once cleaned, the facial portions were detached, and the neurocrania were left to dry at room temperature for 5 days in a well-ventilated area.

The skulls were scanned in 3D using the Shining 3D EinScan SP 3D scanner (Shining3D, Hangzhou, China). The scanning process was performed using a fixed rotary table. After scanning, the obtained data was processed with EXScan Pro software (Shining3D, Hangzhou, China) for mesh operations, and the resulting models were saved in PLY format for further analysis.

**Fig. 1:**
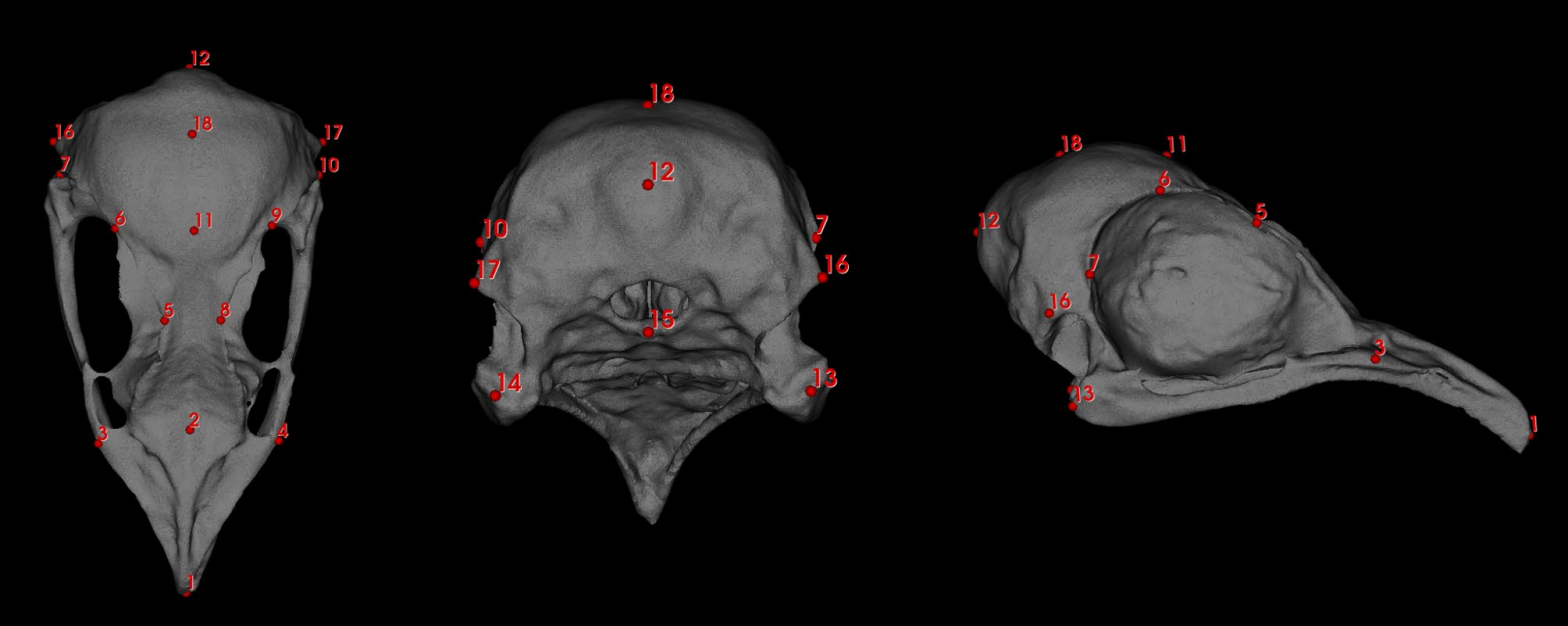
Landmarks: 1 – apex of the premaxilla; 2 -processus frontalis of the premaxilla; 3,4 - most rostral point of the antorbital fenestra; 5, 8 - the most medial point of the supraorbital margin; 6, 9- supraorbital margin on the level of the nasofrontal hinge, 7, 10 – postorbital process; 11 the mid-point of the nasofrontal hinge; 12 – external occipital protuberance; 13, 14 – mandibular process; 15 - the most ventral point of the occipital condyle; 16, 17 - otic process; 18 – the highest point of the braincase.

For the landmarking procedure, 18 landmarks were manually placed on each skull, following anatomical regions identified as relevant in previous studies^22^. Landmarks were positioned to represent key cranial features, avoiding areas of low inter-individual variation to reduce measurement errors (Fig. 1). A single researcher performed all landmarking to ensure consistency. The landmarking was conducted using Slicer software (version 5.2.2).

### Statistical analysis

All statistical analyses were conducted using R software (version 4.0.5). Procrustes Analysis (GPA) was performed to align the landmarks and remove non-shape variation, including translation, scaling, and rotation^12^. The resulting Procrustes coordinates were used for subsequent analyses. The Centroid Size (CS), representing the overall size of the cranial structure, was calculated using the Procrustes-aligned landmarks. The relationship between centroid size and other morphometric variables was analyzed using regression models. Principal Component Analysis (PCA) was performed on the GPA-aligned data to capture shape variation among the specimens^20^. Only the first two principal components (PC1 and PC2) were considered, as the remaining components explained less than 10% of the variation and were excluded from the analysis. The significance of differences in PC values and centroid size between males and females was tested using independent-sample t-tests, and the data were analyzed using Procrustes ANOVA to assess overall shape variation^23^. P-values were adjusted for multiple comparisons using the Bonferroni correction when necessary. Regression analysis (RA) was used to investigate the relationship between cranial shape and body weight and length variables. Linear regression was used to model the relationship between the principal components and the morphometric variables^24^.

## Results

### Shape and size

The shape analysis results (Table 1) indicated that sex has a statistically significant effect on shape (F = 1.8286, *P* = 0.039). The proportion of variance explained was 1.83%, with an effect size of Z = 1.7643, suggesting a significant difference in skull shape between males and females, which is in agreement with the results from Owens and Hartley^10^. In contrast, centroid size analysis revealed that sex does not significantly affect skull size (F = 0.0021, *P* = 0.972). The proportion of variance explained was extremely low (0.002%), with an effect size of Z = -1.9382, indicating that male and female individuals have similar overall skull sizes, aligning with Fairbairn’s (1997) observations on allometry in sexual size dimorphism. Fairbairn,^25^.

For female individuals, the effect of centroid size on shape was not statistically significant (F = 1.2234, *P* = 0.233). The proportion of variance explained was 2.18%, with an effect size of Z = 0.73104, indicating that centroid size does not significantly affect skull shape in females^26^. A similar pattern was observed for male individuals, with centroid size not having a significant effect on shape (F = 0.999, *P* = 0.429). The proportion of variance explained was 2.38%, with an effect size of Z = 0.16309, confirming that shape in males is also not correlated with centroid size^26^.

**Table 1:** ANOVA results for shape and centroid size.

Effect	MS	Rsq	F	Z	P
Shape	0.0077474	0.01832	1.8286	1.7643	0.039
Size	2.05	0.00002	0.0021	−1.9382	0.972

### Shape variation

**Fig. 2:**
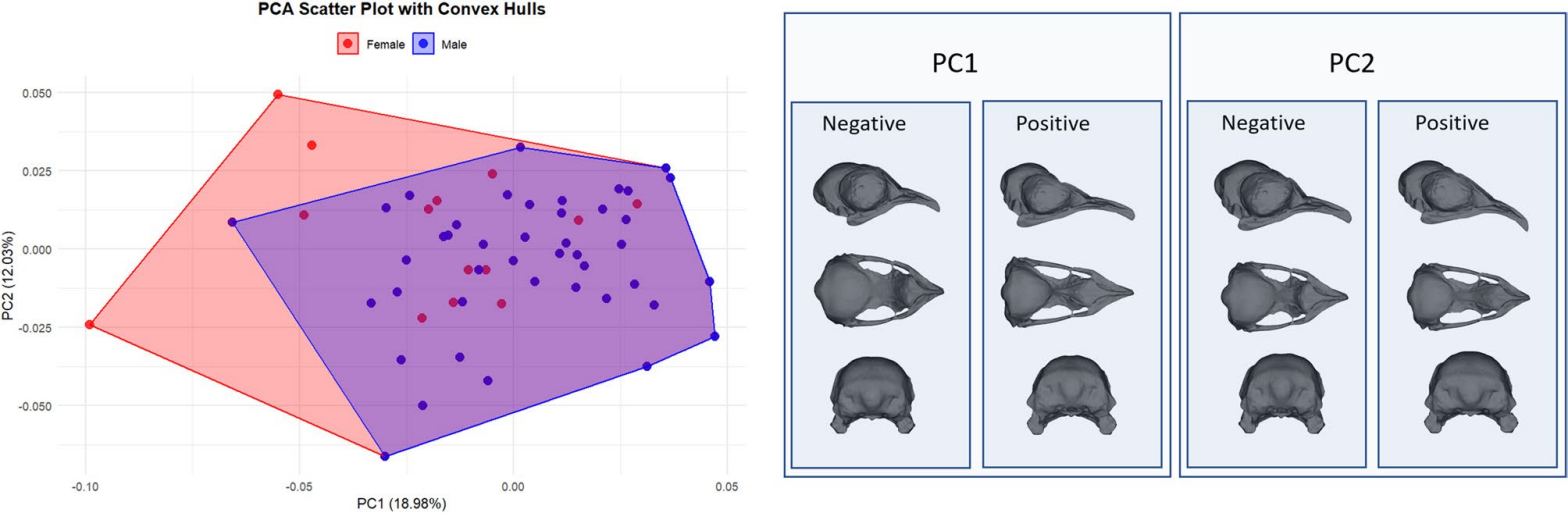
PCA results.

Analysis of PC1 and PC2 values (Fig. 2) revealed that males have a slightly positive mean PC1 value (+ 0.0031), whereas females have a somewhat negative mean (-0.0029), suggesting a potential morphological difference^13^. However, the minimum and maximum values are similar between sexes, indicating substantial distribution overlap. For PC2, females have a slightly higher mean (-0.0019) than males (-0.0042), indicating a minor shift towards more negative PC2 values in males. However, similar first and third-quartile values suggest that PC2 does not differentiate males and females strongly.

Although Procrustes ANOVA identified a significant shape difference between males and females, PC1 and PC2 do not fully capture this distinction^23^. This may be because PC1 and PC2 account for only a portion of the total variance, and sex-related shape differences may be more prominent in other components. Procrustes ANOVA evaluates overall shape differences, while PCA highlights directions with the highest linear variance, potentially missing complex patterns^27^.

The analysis identified PC1 as the most discriminative principal component between sexes. However, statistical tests revealed no significant difference in PC1 values between the sexes (t = -1.1412, df = 96.086, *p* = 0.2566). The 95% confidence interval (- -0.0167 to 0.0045) includes zero, suggesting that the observed difference may be due to random variation rather than an actual morphological distinction^11^. The mean PC1 value for females was − 0.00299, while for males, it was 0.00310, indicating a slight but non-significant shift^28^.

### The influence of weight and cranium length on skull shape

**Fig. 3:**
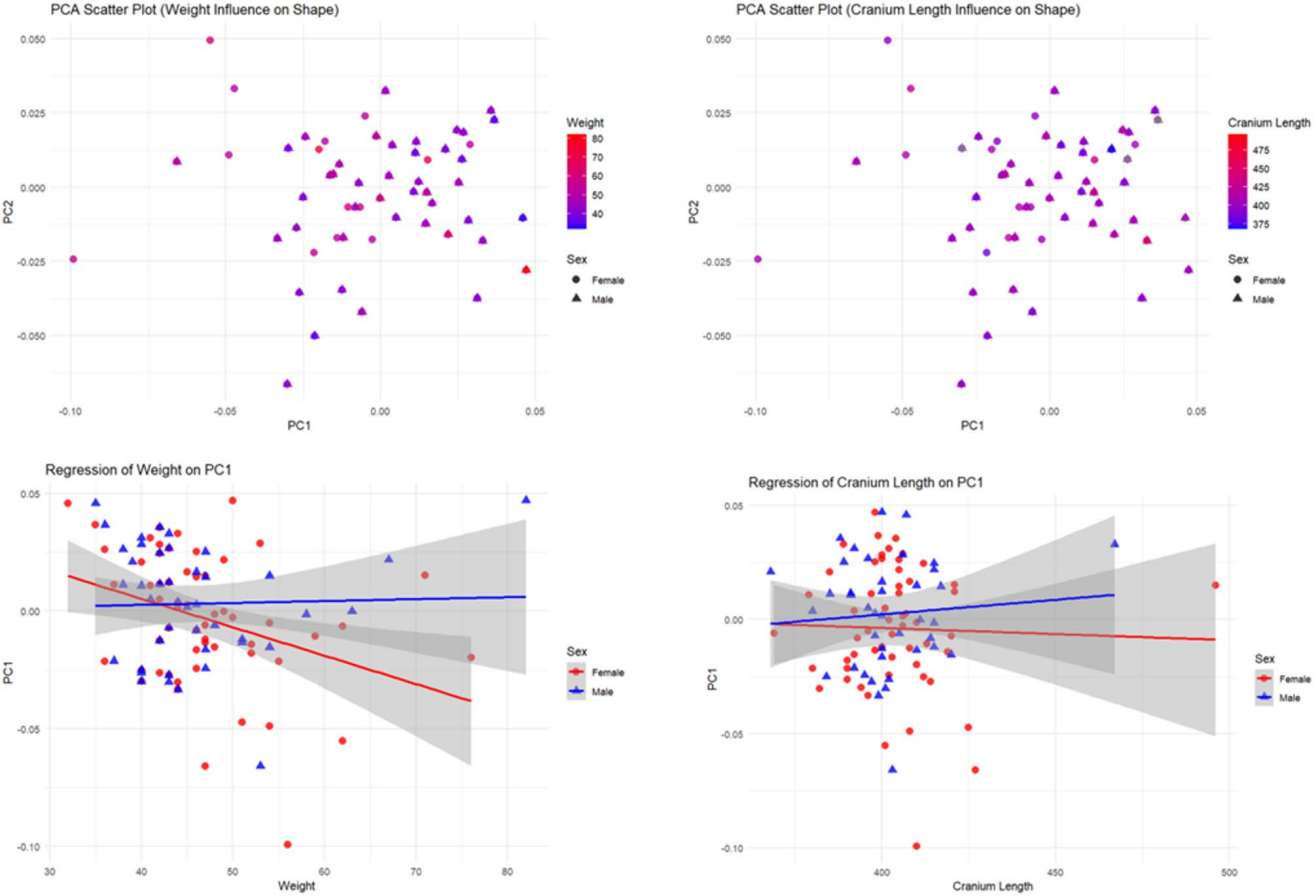
The influence of weight and cranium length on skull shape: PCA and RA Top row: PC1–PC2 scatterplots colored by (left) body weight and (right) cranium length; symbols indicate sex. Bottom row: sex-specific regressions of PC1 on (left) body weight and (right) cranium length, with shaded confidence bands.

Procrustes ANOVA results indicate that weight significantly affects skull shape in females (F = 2.1322, *P* = 0.018) and males (F = 3.033, *P* = 0.004). The proportion of variance explained is higher in males (6.89%) than in females (3.73%), with a stronger effect size (Z = 2.7302 vs. Z = 2.0511), suggesting more pronounced shape changes with increasing weight in males^14^. Regression analysis further revealed that body weight influences the relative positioning of landmarks associated with the beak and orbit, with males showing a stronger correlation between weight and rostro-caudal skull elongation (R²=0.0689, *P* = 0.004) compared to females (R²=0.0373, *P* = 0.018). The PCA scatterplots (upper panels) provide complementary context by illustrating how individuals are distributed in morphospace as weight varies. Cranial length, however, does not statistically affect skull shape in either females (F = 1.2685, *P* = 0.214) or males (F = 1.0686, *P* = 0.367), with low variance explained (2.29% in females, 2.73% in males), indicating that cranial length is not a key factor driving morphological changes^15^. This lack of association is also evident in the regression plot (Fig. 3, lower right), where slopes are close to zero for both sexes.

## Discussion

This study provides a comprehensive analysis of cranial morphology in juvenile alpine swifts, utilizing 3D scanning and geometric morphometric techniques to explore the influences of sex, centroid size, body weight, and cranial length^22,29^. These methods enable precise quantification of shape variation through landmark-based analyses, offering a robust framework for studying morphological differences^30^. The development of these techniques over the past decades has provided critical tools for understanding morphological evolution in vertebrates^31^. Recent advances in geometric morphometrics have further enhanced the ability to dissect complex shape variations, providing a foundation for the analytical approaches used in this study^32^. The results reveal subtle sexual dimorphism in skull shape, a significant influence of body weight, and minimal size and cranial length effects. These offer insights into early developmental processes in a species adapted for an aerial lifestyle^33^.

Procrustes ANOVA indicates that sex significantly affects skull shape (F = 1.8286, *P* = 0.039), with a modest proportion of variance explained (1.83%) and a moderate effect size (Z = 1.7643), consistent with early sexual dimorphism in other avian species^10^. In adult alpine swifts, cryptic sexual dimorphism is evident, with males exhibiting longer tail forks for enhanced maneuverability and females showing slightly higher body mass (~ 1%), potentially linked to reproductive demands^3^. The subtle juvenile shape dimorphism observed here may represent an early ontogenetic precursor to these adult traits, particularly male cranial elongation along PC1, which is related to aerodynamic performance. These differences may arise from genetic or hormonal factors initiating developmental divergence^34^. The low variance suggests that sex is not the primary driver of shape variation, with environmental or nutritional influences likely playing a more significant role^16,35,36^. Similar findings in Japanese quails support the applicability of these methods to swifts^13^.

Centroid size analysis revealed no significant effect of sex on skull size (F = 0.0021, *P* = 0.972), with negligible variance explained (0.002%) and a negative effect size (Z = -1.9382), consistent with uniform size in aerial birds supporting flight efficiency^1^. This suggests that size differences, if they emerge, may develop later due to post-fledging growth or sexual selection pressures^5^. The lack of size variation underscores the importance of shape over size in early morphological differentiation^6^. Comparable results in cat skulls reinforce this pattern^8^.

The relationship between centroid size and skull shape was not significant in either females (F = 1.2234, *P* = 0.233) or males (F = 0.999, *P* = 0.429), with low variance explained (2.18% and 2.38%) and weak effect sizes (Z = 0.73104 and Z = 0.16309), indicating that shape variation is mainly independent of size^26^. This decoupling may reflect constrained growth in juveniles, where allometric effects are not yet pronounced^37^. Environmental factors, such as nutritional availability or genetic predispositions, could drive these shape differences^16^. Morphometric studies on sheep skulls similarly found shape variation independent of size^17^.

PCA results showed subtle shape differences, with males exhibiting a slightly positive mean PC1 value (+ 0.0031) and females a slightly negative mean (-0.0029). However, no significant difference was found (t=-1.1412, *P* = 0.2566), with the confidence interval including zero, suggesting random variation rather than robust dimorphism^28^. For PC2, females had a slightly higher mean (-0.0019) than males (-0.0042), but overlapping quartile ranges indicate minimal separation. The limited ability of PC1 and PC2 to capture sex-related differences may stem from their focus on linear variance, missing complex patterns (Klingenberg^27,38^. Advanced morphometric techniques, such as those incorporating linear discrimination and regression analyses, could further elucidate subtle shape variations by visualizing selection gradients^39^. Similar challenges were noted in studies of livestock guardian dog skulls^9^. Body weight changes with age in juvenile alpine swifts, increasing during the nestling phase before stabilizing^40^. The stronger weight-shape correlation observed in males (R² = 0.0689) could therefore reflect age-related heterochrony rather than mass per se, with older (heavier) males exhibiting more pronounced elongation along PC1. Future studies should incorporate direct age proxies (e.g., telomere length or growth bars) to disentangle these effects.

Gündemir’s work on cranial morphology in other species provides a comparative framework, demonstrating similar geometric morphometric approaches to detect shape variations independent of size in mammals such as cats^8^, livestock guardian dogs^9^, sheep^17^, and canids^18^. These studies highlight influences of weight and breed on skull elongation, paralleling our findings in swifts and supporting the decoupling of shape from size across taxa.

Body weight significantly influenced skull shape, with a stronger effect in males (F = 3.033, *P* = 0.004; R²=6.89%; Z = 2.7302) than females (F = 2.1322, *P* = 0.018; R²=3.73%; Z = 2.0511), suggesting greater sensitivity to biomechanical or growth-related pressures in males^41^. In swifts, weight-related adaptations may influence cranial features linked to muscle attachment or aerodynamic performance^42^. Geometric morphometric analyses of avian skulls have similarly shown that biomechanical factors can drive significant shape variation, particularly in species with specialized lifestyles like swifts^43^. This sex-specific response could indicate differential developmental strategies^37^. Comparable weight-driven shape changes were observed in red fox and golden jackal mandibles^18^.

Cranial length showed no significant effect on shape in either females (F = 1.2685, *P* = 0.214) or males (F = 1.0686, *P* = 0.367), with low variance explained (2.29% and 2.73%), suggesting that skull elongation does not drive morphological variation in juveniles^42^. The prominence of weight over length may reflect broader physiological implications of body mass^24^. Similar patterns were noted in sheep skulls^17^.

These findings highlight the complexity of cranial morphology in juvenile swifts, with subtle shape dimorphism and a significant influence of body weight, particularly in males. The absence of size dimorphism and the limited role of cranial length suggest that early developmental processes prioritize shape adaptations over size changes, aligning with the demands of flight^1^. The more substantial weight effect in males may point to sex-specific developmental trajectories, warranting comparison with adults^33^. Gundemir’s work on cranial morphology in other species provides a comparative framework^8,9,17,18^.

The focus on juveniles limits the scope to a specific life stage, and extending analyses to adults could clarify how dimorphism and weight effects evolve. Higher-order components or alternative methods, such as discriminant function analysis or machine learning-based morphometrics, could reveal finer patterns^44^. Environmental variables, such as diet or flight duration, could elucidate non-size-related factors influencing shape^45^. Histological or genetic analyses could uncover mechanisms underlying shape differences^19^. Comparative studies on quails and sheep suggest that combining morphometric and ecological data enhances understanding of developmental processes^13,17^.

## Conclusion

This study demonstrates that in juvenile alpine swifts, skull shape is subtly influenced by sex and significantly by body weight, while size and cranial length play minimal roles^6,14^. These findings provide a foundation for understanding cranial development and underscore the value of combining 3D morphometrics with ecological and developmental perspectives^22,33^. Future research could explore ontogenetic trajectories, environmental influences, and the adaptive significance of cranial morphology in swifts, contributing to a deeper understanding of avian evolutionary biology^46^.

## Data Availability

The datasets used and/or analysed during the current study available from the corresponding author on reasonable request.
